# Overlapping Neuroimmune Mechanisms and Therapeutic Targets in Neurodegenerative Disorders

**DOI:** 10.3390/biomedicines11102793

**Published:** 2023-10-14

**Authors:** Fabiola De Marchi, Ivana Munitic, Lea Vidatic, Eliša Papić, Valentino Rački, Jerneja Nimac, Igor Jurak, Gabriela Novotni, Boris Rogelj, Vladimira Vuletic, Rajka M. Liscic, Jason R. Cannon, Emanuele Buratti, Letizia Mazzini, Silva Hecimovic

**Affiliations:** 1 Department of Neurology and ALS Centre, University of Piemonte Orientale, Maggiore Della Carità Hospital, Corso Mazzini 18, 28100 Novara, Italy; fabiola.demarchi@uniupo.it; 2 Laboratory for Molecular Immunology, Department of Biotechnology, University of Rijeka, R. Matejcic 2, 51000 Rijeka, Croatia; ivana.munitic@biotech.uniri.hr; 3 Laboratory for Neurodegenerative Disease Research, Division of Molecular Medicine, Ruder Boskovic Institute, 10000 Zagreb, Croatia; lea.vidatic@irb.hr; 4 Department of Neurology, Clinical Hospital Center Rijeka, 51000 Rijeka, Croatia; elisha.papic@uniri.hr (E.P.); valentino.racki@uniri.hr (V.R.); vladimira.vuletic@uniri.hr (V.V.); 5 Department of Neurology, Faculty of Medicine, University of Rijeka, 51000 Rijeka, Croatia; 6 Department of Biotechnology, Jozef Stefan Institute, SI-1000 Ljubljana, Slovenia; jerneja.nimac@ijs.si (J.N.); boris.rogelj@ijs.si (B.R.); 7 Graduate School of Biomedicine, Faculty of Medicine, University of Ljubljana, SI-1000 Ljubljana, Slovenia; 8 Molecular Virology Laboratory, Department of Biotechnology, University of Rijeka, R. Matejcic 2, 51000 Rijeka, Croatia; igor.jurak@biotech.uniri.hr; 9 Department of Cognitive Neurology and Neurodegenerative Diseases, University Clinic of Neurology, Medical Faculty, University Ss. Cyril and Methodius, 91701 Skoplje, North Macedonia; gabriela.novotni@medf.ukim.edu.mk; 10 Faculty of Chemistry and Chemical Technology, University of Ljubljana, SI-1000 Ljubljana, Slovenia; 11 Department of Neurology, Sachsenklinik GmbH, Muldentalweg 1, 04828 Bennewitz, Germany; rajka.liscic@sachsenklinik.de; 12 School of Health Sciences, Purdue University, West Lafayette, IN 47907, USA; cannonjr@purdue.edu; 13 Purdue Institute for Integrative Neuroscience, Purdue University, West Lafayette, IN 47907, USA; 14 International Centre for Genetic Engineering and Biotechnology (ICGEB), Padriciano 99, 34149 Trieste, Italy; emanuele.buratti@icgeb.org

**Keywords:** Alzheimer’s disease, Parkinson’s disease, Niemann–Pick type C disease, neurodegeneration, neuroinflammation, immunomodulatory therapies, rare diseases

## Abstract

Many potential immune therapeutic targets are similarly affected in adult-onset neurodegenerative diseases, such as Alzheimer’s (AD) disease, Parkinson’s disease (PD), amyotrophic lateral sclerosis (ALS), and frontotemporal dementia (FTD), as well as in a seemingly distinct Niemann–Pick type C disease with primarily juvenile onset. This strongly argues for an overlap in pathogenic mechanisms. The commonly researched immune targets include various immune cell subsets, such as microglia, peripheral macrophages, and regulatory T cells (Tregs); the complement system; and other soluble factors. In this review, we compare these neurodegenerative diseases from a clinical point of view and highlight common pathways and mechanisms of protein aggregation, neurodegeneration, and/or neuroinflammation that could potentially lead to shared treatment strategies for overlapping immune dysfunctions in these diseases. These approaches include but are not limited to immunisation, complement cascade blockade, microbiome regulation, inhibition of signal transduction, Treg boosting, and stem cell transplantation.

## 1. Introduction

Neurodegenerative diseases (NDDs) share several common mechanisms, the most prominent of which is neuroinflammation [1,2]. Neuroinflammation, evident as the activation of microglia and astrocytes, which results in increased proinflammatory cytokine and reactive oxygen species generation, is one of the main mechanisms that cause neuronal death. Although it was traditionally considered to be almost exclusively a late step in disease pathogenesis, multiple lines of evidence have recently shown that it could be an early step as well (reviewed in [3,4]}. This review focuses on overlapping neuroimmune mechanisms and potential shared therapeutic targets in several key NDDs, which is important because the vast majority of patients with adult-onset NDDs lack Mendelian genetic risk factors that can be targeted by gene therapies. In a recent review article, we already discussed the proposed immune imbalance underpinning amyotrophic lateral sclerosis (ALS) and frontotemporal dementia (FTD) [5], which we extend here to two other late-onset NDDs—Alzheimer’s disease (AD) and Parkinson’s disease (PD)—and an early-onset Niemann–Pick type C disease (NPC) due to its extensive and intriguing overlap with AD. We aimed to depict common targets that could lead to faster translation and shared therapies.

It is of note that during the past decade, we have witnessed an impressive breakthrough in the treatment of multiple sclerosis (MS) [6], resulting in clinically meaningful changes in the disease course, something that is still inconceivable in AD, PD, ALS, and FTD. In contrast to the latter, MS is an autoimmune disease of the CNS, whose relapsing–remitting form is rather efficiently controlled by various immunomodulatory and immunosuppressive treatments, especially those targeting T and B cells [7,8]. However, in progressive forms of MS, which account for up to 15% of cases, an increase in neurological disability is not prevented since chronic inflammation and neurodegeneration advance despite immunosuppressive or immunomodulatory treatment. Although MS is not extensively covered in this review, we draw several parallels to the neurodegenerative aspect of MS and pinpoint some common mechanisms/targets between progressive MS and classical NDDs.

### Brief Overview of Distinct and Overlapping Clinical Features in Neurodegenerative Diseases

AD is a progressive degenerative disease of the brain and the most common cause of dementia among elderly people, accounting for at least two-thirds of all dementia cases [9]. AD is defined as a progressive decline in cognitive function, typically beginning with memory impairment, and a characteristic change in personality and executive functions. FTD represents a group of disorders considered to be clinically and pathologically distinct from AD, although FTD may be mistaken for AD in the early clinical stages [10,11]. FTD clinically presents as either behavioural or aphasic variants, reflecting the topography of the underlying synaptic and neuronal loss [10,12,13]. The most common behavioural or frontal variant of FTD is associated with disinhibition, impulsivity, apathy, and loss of insight, which disturb social interaction, and it is typically accompanied by marked frontal lobe atrophy. The aphasic variant is further divided into two subtypes: the non-fluent form (primary progressive aphasia) with hesitant diminished speech output, for which left frontotemporal lobe involvement is characteristic, and the fluent form (semantic dementia) with severe deficits in naming, word comprehension, and visual recognition (agnosia) of faces and objects that involves the bilateral anterior temporal lobes. Therefore, the clinical phenotype of FTD may overlap with AD in memory and executive dysfunction, but it is distinct in terms of behavioural problems and language difficulties. ALS and FTD are related clinical phenotypes that are characterised by a decline in motor, cognitive, and behavioural function and short survival. ALS is the most common adult-onset motor neuron disease, characterised by progressive, irreversible motor neuron loss leading to the denervation-mediated atrophy of muscles and death by respiratory failure. However, it is increasingly recognised that ALS is a multisystem disorder in which other non-motor (cognitive and behavioural) impairments can be observed, whereas, on the other side, FTD can be associated with signs of motor neuron disease (FTD-MND) [14]. 

Niemann–Pick type C disease (NPC) is an autosomal recessive neurovisceral lipid storage disorder characterised by liver dysfunction and progressive neurodegeneration [15,16]. It is characterised by a highly heterogeneous and variable clinical phenotype, from a rapidly progressing neonatal form to an adult-onset chronic neurodegenerative condition. The neuropathological features of NPC disease include the loss of Purkinje neurons in the cerebellum, the hyperphosphorylation of tau and the widespread occurrence of neurofibrillary tangles (NFTs), the presence of dendritic and axonal abnormalities, and profound neuroinflammation (activated astrocytes and microglia) [17,18,19,20]. While hepatosplenomegaly together with motor problems are initially observed in neonatal and infantile forms along with other symptoms, the juvenile and adult forms of NPC are typically manifested by a variety of progressive neurological and/or psychiatric symptoms, including ataxia, dystonia, hearing loss, epileptic seizures, dysarthria, dysphagia, cognitive impairment, and dementia. Indeed, these NPC patients display an apolipoprotein E ε4-dependent accumulation of amyloid-ß (Aß) peptides into diffuse Aß plaques as well as the widespread occurrence of NFTs in their brains [17,18,19], the two characteristic features of AD. For this reason, NPC disease is often called juvenile AD.

PD is the second most common NDD after AD, the cardinal motor features of which include akinesia/bradykinesia, postural instability, and resting tremor. Here, it should be noted that while many of the motor symptoms arise from the loss of dopamine neurons in the substantia nigra, neuropathology occurs systemically and elsewhere in the brain, resulting in an array of additional motor and nonmotor symptoms; notably prominent are constipation (damage to the enteric nervous system), mental health effects, rapid eye movement (REM) behaviour disorder, and loss of cognitive function, which can be especially prominent at the late disease stages [21,22]. The affected functions vary from defects in performing executive tasks, visual perception, attention, memory loss, and dementia. Cognitive impairment has been reported in up to 90% of PD cases, with dementia cases comprising up to 30% of cases, thus making cognitive manifestation one of the most important non-motor aspects of the disease. Overall, it has become clear that NDDs often show broadly mixed pathologies and that many patients are now considered to belong to a disease spectrum rather than a discrete NDD. 

The importance of all of these overlapping features of NDDs should be more appreciated, especially in cohort characterisation for clinical studies, the development of future therapies, biomarker design, and monitoring, with the ultimate goal of precision/personal medicine.

## 2. Overlapping Pathogenic Mechanisms in Neurodegenerative Diseases

**Figure 1:**
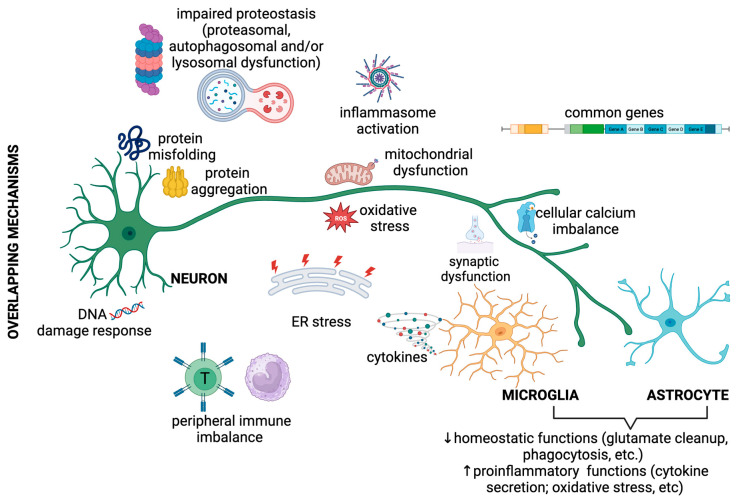
Common disease mechanisms of the most investigated neurodegenerative diseases. Among the main involved mechanisms, there are factors related to impaired proteostasis (resulting from impaired protein folding and/or defects in protein degradation by proteasomes and autophagy), metabolic stress (mitochondrial dysfunction and oxidative stress), and neuroinflammation (inflammasome activation and proinflammatory cytokine secretion). The processes in the CNS are commonly linked to peripheral immune imbalance. Crosstalk between proteinopathy and immune cell activation is present at multiple levels and between multiple cell types, as further detailed in the text, and can result in a vicious cycle that leads to motor neuron death. Of note, very few common genetic risk factors have been linked to neurodegenerative diseases.

The pathogenic mechanisms implicated in NDDs are not linked to individual clinical entities. By contrast, NDD pathogenesis shows considerable overlap in protein misfolding and aggregation; defects in the endosomal–lysosomal network and the clearance of damaged proteins by autophagy or proteasomes; mitochondrial dysfunction and oxidative stress; inflammasome dysfunction; cellular calcium imbalance; impaired axonal, membrane, or nucleocytoplasmic trafficking; DNA damage response; and synaptic dysfunction, many of which crosstalk and are directly or indirectly linked to neuroinflammation and even systemic (peripheral) immune imbalance (Figure 1) [22,23,24,25,26,27,28,29,30,31,32,33]. Most of these mechanisms are also affected by ageing, the most prominent risk factor for adult-onset NDDs, which is, in the immune system, linked to immunosenescence, with increased activated adaptive immune cells and a decreased repertoire of naïve cells, and chronic low-grade inflammation [34,35]. In this chapter, we focus on overlapping proteinopathies and immune imbalance in NDDs, the two features that have recently been tackled for designing and monitoring efficient therapies.

### 2.1. Overlapping Proteinopathies

Thanks to our increased ability to detect pathological protein species in the brains of patients affected by NDDs, it has been clear for the past decade that overlapping proteinopathies exist throughout the entire spectrum of most NDDs [36]. Notably, Aβ aggregates are commonly present in AD and NPC; tau is commonly present in AD, NPC, and FTD; TDP-43 is commonly present in ALS and FTD; and α-synuclein (α–syn) is commonly present in PD [17,18,19,23]. Less commonly, but by no means as an exception, TDP-43 pathology is found in AD and PD, Aβ pathology is found in PD, and α-syn pathology is found in AD and FTD. More recently, it was described by some of us that purified prion protein (PrP) aggregates seeded in cells can convert soluble TDP-43 in non-dynamic protein assemblies with the consequent loss of TDP-43 splicing regulation in the nucleus [37]. The presence of α-syn-positive Lewy bodies, tau, and TDP-43 pathology in a recently described case of sporadic Creutzfeldt–Jakob disease (sCJD) shows that the co-occurrence of multiple proteinopathies is a growing reality in neurodegeneration research and the clinic [38]. 

Just to provide some indication of how frequent comorbidities may be in neurodegeneration, it was estimated that only 20% of all AD cases that occur after the age of 70 can be defined as “pure” AD cases [39]. In most of these cases, the comorbidity occurs between tau pathology and another major actor in NDDs represented by the TDP-43 protein, which plays a major role in ALS and FTLD-TDP, as recently reviewed by Riku et al. [40]. The occurrence of comorbidities has substantially changed our view of neurodegeneration processes from considering AD, PD, ALS, FTD, etc. as distinct and well-defined pathologies to viewing them as a potentially interconnected spectrum of neurodegeneration, in which comorbidities may often influence the main pathology and affect both disease progression and duration. When trying to address this new finding, another important question to answer is whether the overlapping pathologies have the same site-specific characteristics as when they are alone. For both questions, some answers have already been provided with regard to prominent comorbidities. For example, TDP-43 pathology in AD brains considerably differs from the primary motor cortex involvement that is characteristic of this protein in ALS. Rather, TDP-43 pathology in AD brains starts in the amygdala and passes through several stages to finally reach the basal ganglia and middle frontal cortex [41].

Another type of comorbidity that has been studied in the past, especially at the mechanistic level, is represented by α-syn in brain inclusions of AD patients, which has been recently reviewed by [42,43]. At the mechanistic level, α-syn and tau have been shown to be connected in several ways, with α-syn fibrils being able to promote tau aggregation [44,45]. Taken together, these observations suggest that protein aggregation comorbidities can play an important role in NDDs and that their study could be prioritised in future studies to better understand their pathological and clinical connections.

In addition, the careful identification of comorbidities could also be pivotal for the interpretation of clinical trial results. In fact, through the development of appropriate biomarkers, it would be greatly advantageous to start stratifying treated subjects by the presence and types of accompanying comorbidities. This action might be able to uncover clinical response variability in some groups compared with others, potentially “rescuing” treatments that might otherwise fail completely if this factor is not considered. For this reason, accurate in vivo comorbidity detection methods are urgently needed for the future of therapeutic research.

Another important area of future research is to investigate the mechanistic triggers that lead to these different protein aggregation profiles. In this respect, there are several possibilities that should be considered. Notably, it is now clear that the protein–protein interactions of the proteins involved in neurodegeneration can change depending on cell type, cell composition, and stress level. This, together with the fact that, under pathological conditions, proteins involved in neurodegeneration mislocalise and alter their protein–protein interactions, could be an important driver of aggregation. For example, the RNA-binding protein ELAV-like protein 4 (ELAVL4), also known as HuD, has been shown to co-localise with phosphorylated TDP-43 and FUS proteins [46,47], both of which participate in the splicing regulation of selected exons in neurons in which ELAVL4 is predominantly expressed [48]. Presumably, therefore, a transiently close interaction during a functional event might favour the co-precipitation of both proteins during aggregation-promoting conditions. Alternatively, it has recently been shown using cryoEM techniques that fibrils of TDP-43, tau, a-syn, Ab, and TMEM106B can adopt several different folds in neurons from patients’ brains (recently reviewed by Scheres et al. [49]). It is thus tempting to speculate that these different folds might also have different abilities to induce the aggregation of additional proteins, which would explain the specific co-aggregation profiles in selected cells or following specific triggers. In addition, it is important to note that TDP-43 has previously been shown to interact with Aβ and that TDP-43 oligomers affect the conformational change in Aβ as well as influence Aβ fibrilisation [50]. Furthermore, TDP-43 also interacts with α-syn, and this interaction has been shown to be synergistic, leading to enhanced mutual aggregation into fibrils [51]. Another possibility to explain the co-aggregation is the observation that proteins such as TDP-43 can induce changes in specific cellular factors, such as hnRNP A1, to promote the production of aggregate-prone forms of these targets that then co-aggregate with the TDP-43 pathology [52]. Finally, a possible explanation for coaggregation, along with different aggregation profiles in NDDs, could also be related to posttranslational modifications (PTMs) of proteins involved in neurodegeneration [53]. PTM homeostasis is disrupted in NDDs, and there is growing evidence that PTMs may be related to the phenotypic diversity of NDDs [53,54,55,56]. For instance, some TDP-43 PTMs are specific to FTLD-TDP type A (associated with GRN mutations) and some are specific to type B (associated with C9orf72 mutations) [55], phosphorylated Tyr526 FUS is present in the FTLD-FUS pathology [54,56], and the acetylation of K280/K281 in tau increases aggregation in AD. However, further research is needed to fully understand the role of PTMs in protein aggregation comorbidities.

Taken together, these observations suggest that protein aggregation comorbidities can play an important role in NDDs and that their study could be prioritised in future studies to better understand their pathological and clinical connections. In discussing the overlapping pathologies in NDDs, we should also reconsider our understanding of protein aggregation as merely pathogenic, as it may be an epiphenomenon or even a protective and compensatory mechanism to cellular stress involved in delaying cell death [57]. It could also be a late stage of neuropathology and could even be absent in rare NDD cases, such as in PD patients carrying *LRRK2* mutations [58,59].

### 2.2. Overlapping Immune Imbalance 

As mentioned above, many pathogenic mechanisms in NDDs are intricately linked to neuroinflammation and systemic immune imbalance. Key cells that respond to neuronal stress are microglia, the resident macrophages in the brain and spinal cord, which are the primary immunocompetent cells in the CNS [60,61]. In response to protein aggregation and various other neurotoxic conditions, microglia dynamically react in different ways, which range from various homeostatic functions that provide neuroprotection (phagocytosis, trophic support, etc.) to damage-associated functions that promote neurotoxicity [61]. Similarly, astrocytes, the most abundant CNS glial cells, modify their functional status in pathological conditions [62]. Protein aggregates of TDP-43 and α-syn have been shown to directly damage mitochondria, causing oxidative stress and/or energy deficits [63,64,65]. The subsequent release of mitochondrial DNA and cellular stress, in turn, lead to the activation of proinflammatory signalling pathways mediated by transcriptional factors nuclear factor kappa B (NF-κB) and interferon regulatory factor 3 (IRF3), along with the activation of the NLRP3 inflammasome [66]. Inflammasomes are also directly activated by tau oligomers or monomers and by lysosomal damage and subsequent cathepsin B release upon the phagocytosis of Aβ aggregates by microglia [67]. It is not surprising then that inflammasome dysregulation has been implicated in the progression of all major neurodegenerative disorders, such as AD, PD, Huntington’s disease, ALS, and prion diseases [67]. Therefore, reactive microglia and astrocytes promote the release of immune mediators, including proinflammatory cytokines and chemokines; decrease the production of protective factors (growth factors, fractalkine, etc); and promote glutamate toxicity, all of which can promote the onset and progression of NDDs and thus represent potential therapeutic targets [68,69]. Notably, progressive MS is marked by similar activation of microglia [70,71].

Several genes directly affecting immune function have been linked to ALS and/or FTD, such as *C9ORF72*, *TBK1*, *OPTN*, *CYLD*, and *GRN* (reviewed in [3,4,5,72]). Moreover, as detailed below, genes enriched in microglia have been increasingly implicated in AD pathogenesis over the past decade. It is interesting though that there is only moderate genetic overlap between NDDs. A recent large GWAS study found evidence for only eleven shared loci in AD, PD, and/or ALS, which were potentially linked to genes affecting lysosomal or autophagic functions, neuroinflammation, DNA damage response, and oxidative stress [73]. However, despite such a comparably small overlap of individual genes, defective functions across the broad spectrum of NDDs are linked to phagocytosis, lysosomal function, autophagy, inflammatory signalling, the activation of complement, and others [74,75,76,77,78]. Many of these functions, including the activation of complement, inflammatory signalling, and phagocytosis, are beneficial only in a narrow window, and if uncontrolled, they can lead to various harmful effects, including extensive synaptic pruning and bystander cytotoxicity [77,78,79,80,81]. The harmful effects of these factors are not limited to the CNS, and many lead to systemic immune imbalance. Higher neutrophil counts and blood proinflammatory factors are linked to a higher risk of developing ALS, AD, and PD [82,83,84,85].

Given that genetics can explain only a small fraction of NDD cases and that most have a complex environmental component, many environmental factors have been researched, such as infectious diseases, microbiome composition, toxins (pesticides and others), most of which affect immune responses. The proposed link between viral infection and NDD has been studied since the Spanish flu of 1918, which was caused by influenza A virus subtype H1N1 [86]. Since then, multiple viruses have been proposed to increase the risk, such as herpes simplex virus 1 (HSV-1) for AD, retroviruses for ALS, and others [87,88,89,90]. Most recently, a similar risk has been found for SARS-CoV2 and is predicted to have a great impact given the large number of affected individuals in the coronavirus disease 2019 (COVID-19) pandemic [91]. In perhaps the most comprehensive study thus far, exposure to 45 viruses was linked to NDDs in a Finnish cohort, and 22 of these associations were replicated in a UK cohort [92]. While large risk effects, such as those reported between viral encephalitis and AD, were rare, moderately increased risk was very common. Notably, severe cases of influenza and pneumonia were significantly associated with five NDDs (AD, PD, ALS, and vascularised and general dementia). Most of the associations were more strongly linked to NDD one year prior to their diagnosis, but some exposures affected the risk up to 15 years prior to diagnosis. Fittingly, associations with neurotropic viruses were the most common (>80%), and none of the viruses seemed to confer neuroprotection. Similar associations have been reported for severe systemic bacterial infections in AD, as further discussed below [93]. Overall, this strongly supports that CNS and systemic inflammation are linked to NDDs and that preventive vaccines should be pursued more aggressively to avoid not only infections but also their long-term aftermath, including NDDs. 

The role of the gut microbiome in the pathogenesis of NDDs is of great interest since it has been shown that its composition differs in people afflicted with NDD compared with the healthy population [94,95]. Changes in relative abundances of different microbial taxa have been shown to factor into neurodegeneration, going as far as to influence the severity of the symptoms of certain diseases, such as in the case of PD, in which it has been shown that changes in the relative abundance of various taxa can be correlated, either positively or negatively, with motor and non-motor symptom severity [96]. It has been hypothesised that these effects are achieved, among other mechanisms, through the gut–brain axis (GBA). The GBA is a complex bidirectional system operating between the intestines and the brain, and it has been demonstrated in a number of studies that it could drive neurodegeneration in conditions such as AD and PD [97]. So far, several ways in which this system could operate have been described. These include retrograde axonal transport across the vagal nerve [98], microbial metabolites such as short-chain fatty acids (SCFA) [98], serotonin–microbiota interaction [99], tryptophan–kynurenine metabolism [100], and immune signalling [101]. Direct evidence for the involvement of the immune system also came from studying *C9ORF72*, the most common genetic risk factor for both ALS and FTD, which acts in autophagy and endolysosomal pathways to suppress inflammation. Indeed, *C9ORF72* has also been shown to suppress microbiota-induced inflammation in mouse models [102]. Therefore, evidence that the immune signalling linked to microbiota can play an important part in disease pathogenesis is being gathered for many NDDs [103,104].

#### 2.2.1. Immune Imbalance in Alzheimer’s Disease

In addition to the characteristic neuropathological features of AD, amyloid plaques and NFTs, Alois Alzheimer also described enhanced gliosis (the activation of glial cells, microglia, and astrocytes) surrounding senile plaques and affected neurons, thus anticipating the important contribution of the innate immune system to AD pathogenesis. While research on early-onset AD (EOAD) placed Aβ accumulation as a central component and an initiator of the pathological cascade leading to neurodegeneration and neuroinflammation, recent work on late-onset AD (LOAD) implicated that immune dysfunction and neuroinflammation could drive neurodegeneration rather than only being considered a (late) consequence of protein aggregation. This has been strengthened by genome-wide association studies (GWASs) that have identified risk factors of LOAD in genes expressed by microglia (*TREM2*, *CD33*, *CR1*, *INPP5D*, *SPI1*, *BIN1*, *PICALM*, *ABCA7*, *SORL1*, *CD2AP*, and the *MS4A* gene cluster) or crucial for microglial development and function (*PU.1*) [105,106,107,108,109]. Thus, microglial dysfunction became considered a major contributor to AD risk [83], challenging the long-standing Aβ hypothesis, which posits that Aβ is the initial disease trigger, while an excessive inflammatory response of microglia is secondary to the accumulation of Aβ peptides and Aβ plaque formation [110,111]. Interestingly, several of the above-listed GWAS risk factors are functionally linked to microglial phagocytosis and Aβ clearance [112,113,114,115], placing the dysfunction of the immune system in the CNS in the centre of AD pathobiology. Although the loss of homeostatic microglial function and the genesis of disease-associated microglia (DAMs) is a characteristic feature of AD, it is still a matter of debate whether DAMs are “good” or “bad”, i.e., whether microglial activation and neuroinflammation are beneficial (neuroprotective) or detrimental (neurotoxic) for disease progression. Notably, single-nucleus RNA sequencing (snRNASeq) in AD brains recently revealed distinct microglia profiles linked to Aβ and tau-associated pathology [116]. On the other hand, chronically altered microglia in AD could compromise not only phagocytosis but also other physiological functions, including cytokine, chemokine, and growth factor secretion [117,118]. Moreover, the APOE ε4 allele, a major genetic risk factor of LOAD [119], has been functionally linked to reduced Aβ clearance [120,121], implying its direct or indirect role in microglial function. Recent snRNA-seq analyses of frozen AD patients’ brains and preclinical models revealed significantly upregulated expression of ApoE in DAMs and not exclusively in astrocytes, as previously assumed [92], supporting an emerging view that microglia are an important contributor to ApoE biology in the CNS [122,123,124,125,126]. In addition to genetic risk factors that link the genesis of LOAD with microglial dysfunction, ageing is another important risk factor that may influence microglial phagocytic capacity and neuroinflammation through epigenetic mechanisms. Indeed, the age-dependent accumulation of Aβ in LOAD patients seems to be associated with an age-related decrease in microglial phagocytic capacity [127]. Furthermore, a study using an AD mouse model (APP/PS1) showed that only young microglia from wild-type mice cleared Aβ plaques and that exposure of old microglia to conditioned media from young microglia increased their proliferation and reduced Aβ plaque size [127]. This suggests that microglial dysfunction in AD could be reversible and that the phagocytic ability could be modulated to prevent and/or restrict Aβ accumulation. The NLRP3 inflammasome is activated by Aβ, so it has also been implicated as a key component of the innate immune response to proteotoxic stress in AD, triggering pro-inflammatory polarisation and gasdermin D oligomerisation, thus promoting the development of AD pathology [67,128]. Lastly, it is now increasingly accepted that infectious diseases may be involved in the aetiology of AD, as exemplified by the recent influence of COVID-19 on a spectrum of neurological manifestations [129,130,131]. Similarly, other severe infections requiring hospitalisation have recently been shown to increase the risk of dementia [93], further supporting the important role of the immune system in the pathogenesis of AD. 

#### 2.2.2. Immune Imbalance in Niemann–Pick Type C Disease

**Figure 2:**
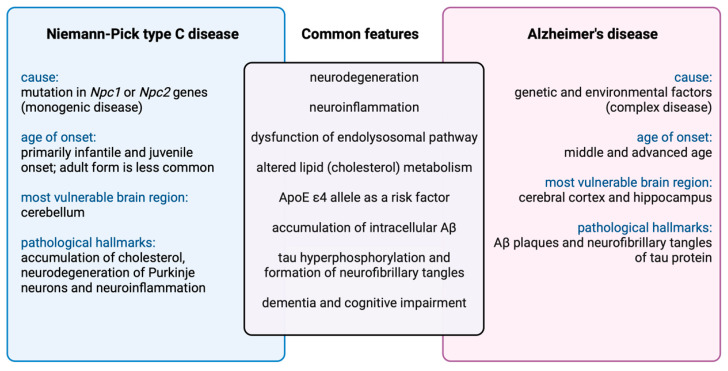
Common features and differences between Alzheimer’s disease and Niemann–Pick type C disease. An especially prominent overlap has been observed between AD and NPC, two disorders with very distinct aetiologies. Created by BioRender.com.

It is intriguing that a rare inherited lysosomal and lipid storage disorder, Niemann–Pick type C disease (NPC), shares several key features with AD [132] (Figure 2). Among these, neuroinflammation seems to play an early and important role in disease progression, together with neurodegeneration. However, in contrast to the complex genetics of AD, NPC is a monogenic disease caused by mutations in the NPC1 and NPC2 genes (95% and 5% of cases, respectively) [132]. These mutations result in the dysfunction of cholesterol transport proteins NPC1 or NPC2 and the accumulation of unesterified cholesterol and other lipids (e.g., glycosphingolipid, sphingomyelin, and sphingosine) in late endosomes/lysosomes and their dysfunction [133,134]. The molecular mechanism of neurodegeneration and neuroinflammation in NPC is currently unknown, but although peripheral organs such as the liver and spleen are also affected, NPC1 expression restricted to the CNS was capable of rescuing both neurodegeneration and lethality in NPC1 null mice [135]. Notably, the restoration of NPC1 in neurons alone does not fully rescue the phenotype, indicating that NPC1 is functionally important in other CNS cells as well [136,137,138,139]. Indeed, NPC1 is ubiquitously expressed throughout the brain, with particularly high expression in microglia and oligodendrocytes [140]. It has been generally assumed that neuroinflammation in NPC is secondary to neuronal loss. However, recent findings in NPC1 mice and NPC patients’ blood-derived macrophages [140] suggest a possible causative rather than consequential role of neuroinflammation in NPC neuropathology. Notably, NPC microglia proteome changes precede neuronal loss and contribute to neuropathology in a cell-autonomous manner. Importantly, lipid accumulation in NPC1 mouse microglia is a consequence of impaired lipid trafficking with a striking accumulation of multivesicular bodies, while lysosomal degradation function seems to be preserved. Among these, the late endosomal/exosomal marker CD63 was the most significantly changed protein in the presymptomatic stage, suggesting that defects within endosomal/lysosomal trafficking and sorting may be among the earliest pathological alterations in NPC microglia. Recently, single-cell transcriptomics of the NPC1 mouse cerebella identified the earliest gene expression changes in microglia cells together with endothelial cells [123], further supporting an important role of microglia dysfunction and neuroinflammation in the pathogenesis of NPC disease. A pathway analysis of differentially expressed genes revealed that activated microglia in NPC1 mice resembled those in an AD mouse model rather than those in an ALS mouse [123].

#### 2.2.3. Immune Imbalance in Parkinson’s Disease

While specific neuronal populations (dopamine neurons in the substantia nigra) are affected in PD, there is also a considerable overlap between AD and ALS in pathogenic mechanisms [27,28,29,30]. In keeping with this, complex interactions have been revealed between α-syn, a neuronal protein associated with PD and other synucleinopathies, and microglia [141]. Like in ALS and AD, microglia are neuroprotective in the early stages of disease by clearing α-syn, whereas during the chronic disease stage, they are considered to promote neurodegeneration by propagating the α-syn burden and creating an inflammatory environment [142]. In addition, viral infections, including COVID-19, have been associated with an increased risk of PD [143], suggesting that inflammation linked to infection could contribute not only to AD but also to PD pathogenesis (as detailed above). Importantly, these findings suggest that neuroinflammation may be a primary pathogenic event and that systemic neuroinflammatory insults may drive the transsynaptic spread of PD pathology. The link between PD, microbiota, and immunity is an important topic. Retrograde axonal transport of pathology across the vagal nerve was hypothesised in 2003 by Heiko Braak using the model of PD [143]. The idea was that α-syn accumulation actually begins in the gut, with pathologic forms of the protein migrating into the brain through the vagal nerve. Evidence of this was shown in 2014, with microtubule-associated transport being a key mechanism [144]. More recently, the same enteric propagation was proposed for β-amyloid in AD. In a mouse model, it was shown that intra-gastrointestinal application of Aβ plaques led to a higher deposition of these plaques in various regions of the brain, with retrograde vagal transport being a key pathway behind it [144]. Microbiota metabolites were also shown to have an effect on the CNS, with short-chain fatty acids (SCFAs) being the most prominent products, affecting the regulation of enteric secretion and motility as well as gut–brain signalling [145]. In various models, it has been shown that SCFAs can have a neuroprotective role, such as in the case of *Lactobacillus plantarum* and its product butyrate, which has anti-inflammatory effects [145]. It also positively influences blood–brain barrier (BBB) permeability [146]. On the other hand, in some cases, SCFAs can have a detrimental effect, such as in the case of propionic acid, in which serum levels were positively correlated with motor and non-motor symptom severity in PD [146]. Besides SCFA, microbiota have also been implicated in taurine metabolism [147] as well as magnetite and hydrogen sulphite production [147], both of which have been shown to play a role in PD. Serotonin is thought to be an important part of GBA signalling, with certain bacteria affecting both its colonic and serum levels, aiding in SCFA production, which in turn increases serotonin production [148]. Microbiota also have an effect on the serotonin precursor tryptophan. It was shown in one study that a sex-dependent increase in hippocampal serotonin levels could be attributed to microbiota-related changes in tryptophan levels [149]. Furthermore, kynurenine, a metabolic product of tryptophan, has been shown to traverse the BBB and lead to neuroinflammation as well as neurodegeneration [100]. Immune signalling linked to microbiota can play an important part in the development of neurodegenerative diseases [103,150]. Alterations in the gut microbiota can lead to changes in the permeability of the intestinal mucus layer, leading to local immune activation and intestinal barrier dysfunction, eventually allowing various microbial triggers, such as lipopolysaccharide (endotoxin) and peptidoglycans to be released into the systemic circulation. This leads to a process known as metabolic endotoxemia, and it has been shown to trigger immune activation in various systems, including the central nervous system, most notably through microglia [151]. Macrophages have also been shown to be intrinsically linked to the gut microbiome. Antibiotic-induced changes in the microbiota have been shown to decrease macrophage levels and affect gastrointestinal motility [151]. With macrophages being essential responders to intestinal injury, this decrease could further enhance the neuroinflammatory effects of metabolic endotoxemia.

There are also direct examples of toxic insults that may influence both PD and ALS. For example, environmental exposures have been extensively studied as risk factors for PD [152]. The organophosphate pesticide chlorpyrifos has been identified as a possible risk factor for PD in both human and animal studies; in addition to inhibiting acetylcholinesterase, it also likely affects dopaminergic neurotransmission and produces oxidative stress [153,154,155,156,157,158,159]. While the role of environmental exposures in ALS is far less understood than that in PD, pesticides have also been explored as risk factors, with some studies suggesting increased ALS risk with pesticide exposure, especially related to organophosphate pesticides, such as chlorpyrifos [160]. Interestingly, mutations in genes specifically responsible for chlorpyrifos detoxification also seem to increase ALS risk, suggesting a gene–environment interaction in the aetiology [161,162]. With respect to genetic overlap, an example is optineurin, which has been extensively studied in ALS and glaucoma [163]; mutations may also be a risk factor in PD [164,165]. While optineurin has many roles (i.e., innate immunity and mitophagy), in vivo experimental PD models, alterations in mitophagy expression, and localisation suggest that these are likely important in both ALS and PD [166]. Taken together, therefore, there is a clear pathogenic overlap between ALS and PD. Overall, there is mounting evidence that the immune system may be a primary target of PD-relevant exposure versus just a downstream pathogenic pathway. For example, a recent line of research has advanced the understating of how environmental agents interact with specific innate immune signalling pathways in microglia to stimulate conversion to a neurotoxic phenotype. Here, researchers showed that NF-κB signalling in microglia is critical to the clearance of aberrant α-syn resulting from rotenone exposure, an important finding that identifies neurotoxin–immune system phenotypic links [166].

## 3. Common Therapeutic Approaches in Treating Immune (Dys)Function in Neurodegenerative Diseases

**Figure 3:**
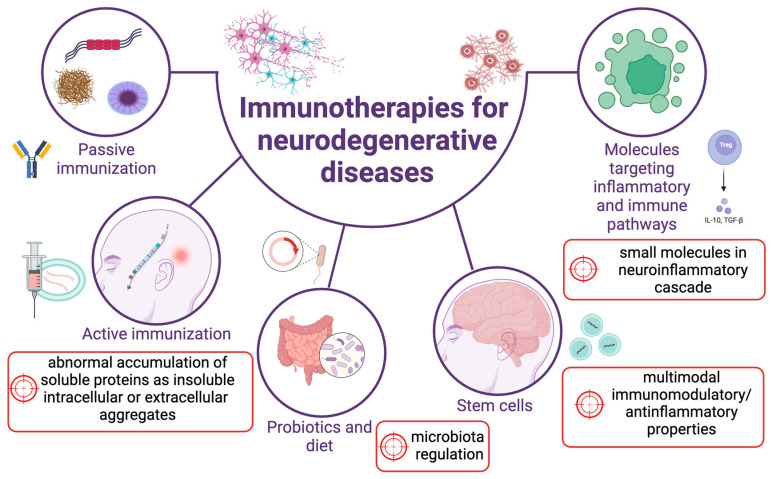
Schematic representation of the main immunomodulatory mechanisms currently being studied as a treatment for neurodegenerative diseases.

Currently, there are no treatments that are able—from a clinical point of view—to invert the course of NDDs, and most therapies are symptomatic [167]. The anti-glutamatergic agent riluzole, the ROS scavenger edaravone, and the ER and mitochondria targeting drug combination PB/TUDCA (sodium phenylbutyrate with taurursodiol) represent rare examples of disease-modifying drugs in ALS. However, these drugs, many of which are not even approved worldwide, only mildly affect the underlying disease cause and course. Notably, the vast majority of therapeutic strategies investigated for NDDs are focused only on single targets. The recent understanding of multiple shared pathologies among NDDs with considerable mechanistic overlap necessitates the development of new, shared, and integrated therapeutic targets. Taking into consideration that (neuro)inflammation and neurodegeneration overlap, coexist, and exacerbate one another in the spectrum of different NDDs, it is tempting to speculate that the neurodegenerative process might be mitigated or even prevented by targeting inflammation. The challenging part is 1) to identify the proper time point for therapeutic intervention, as the chronology of these events varies among different NDDs, and 2) to decipher when the inflammation has a detrimental effect and when it has a protective effect [168]. However, despite the challenges, several emerging findings suggest a promising role of therapies that are able to act on immune dysfunction to slow down the neurodegenerative process. As described above, many reports support the idea that molecules affecting the immune system pathways crosstalk to mechanisms that trigger the misfolding and aggregation of proteins that accumulate in NDDs [4,169,170]. The most currently investigated treatments include active and passive vaccinations, the molecules directly targeting the inflammatory mediators or pathways, and the multimodal effects of stem cells, particularly those of mesenchymal ones (summarised in Figure 3).

### 3.1. Passive and Active Vaccination Therapies Targeting Protein Aggregates

In the past three decades, active vaccinations and monoclonal antibodies for passive vaccinations have been applied in several fields of medicine, including neoplastic and autoimmune diseases. More recently, this therapeutic strategy is also being evaluated for neurological diseases. In most NDDs, the primary underlying pathological mechanism is the abnormal accumulation of soluble proteins in the form of insoluble intracellular or extracellular aggregates, which can act directly as toxic aggregates or via precursors or mediators and on which vaccination therapies can work [171]. More specifically, immunotherapeutic approaches include both passive and active immunisation. The first consists of an infusion of monoclonal antibodies directed against the target molecules (e.g., misfolded proteins); the second provides specific antigens directed towards a specific adaptive immune response [172], inducing the production of antibodies or modulating the inflammatory response. Certainly, the most relevant example is the removal of Aβ accumulation in AD. However, since intracellular protein accumulation is a hallmark of most NDDs, the same approach has also been used for intracellular proteins, α-syn and tau among them. Here, we summarise the main clinical trials recently developed.

#### 3.1.1. Clinical Trials on Aβ Immunisation

Currently, passive immunisation with antibodies against Aβ is the most advanced immunotherapy under investigation. It started from solid preclinical data showing the ability of Aβ-targeting monoclonal antibodies to bind the Aβ-42 species, reducing toxicity, preventing cell death, and restoring plasticity at the hippocampal level in animal models [173,174,175]. Different Aβ-targeting monoclonal antibodies have been proposed in recent years for AD treatment. Due to their different binding properties, aducanumab [176] and lecanemab [177] are among the most promising. Aducanumab was approved after two large trials (EMERGE, 1638 patients, and ENGAGE, 1647) with AD patients in the early stage of the disease [176]. The primary outcome was the changes over treatment in the global cognition (measured with the Clinical Dementia Rating Sum of Boxes test) (CDR-SB) [178], and it was met in the EMERGE trial but not in the ENGAGE trial. However, in both studies, promising results were obtained regarding biomarkers, confirming a dose-dependent reduction in AD marker pathophysiology. On the basis of these results, aducanumab (marketed as Aduhelm) was approved by the U.S. Food and Drug Administration in 2021 under the accelerated approval pathway. On the contrary, the European Medicines Agency withdrew its marketing authorisation application for aducanumab to treat the early stages of AD. Also, for US patients, in July 2023, lecanemab, a humanised IgG1 monoclonal antibody that binds with high affinity to Aβ soluble protofibrils, was approved (marketed as Leqembi). In the phase III clinical trial, the drug achieved its primary outcome (the same as that in the aducanumab trial), and a significant reduction in the brain amyloid burden was reported. Clinical trials on active immunisation for Aβ started in 2000 [179]. The first vaccine tested in humans, called AN1792 (Elan Pharmaceuticals), provided the inoculation of amyloid-β42 peptide with an adjuvant. Although positive effects have been observed in post-mortem neuropathological studies [180], the vaccine had several severe side effects (i.e., meningoencephalitis) and the trial was stopped. Even without clinical efficacy, in a long-term follow-up of surviving patients (14 years) [181], all had only very sparse or undetectable plaques in all regions examined. Subsequent trials in this regard obtained better safety results, but no clinical and biological effects were observed [182].

#### 3.1.2. Clinical Trials on Tau Immunisation

Since intraneuronal aggregates of tau protein have been shown to directly correlate with cognitive decline in AD [183,184], they comprise a potentially even more interesting target than Aβ. In the past decades, several encouraging results have been obtained in experimental mouse models, where tau targeting resulted in a reduction in protein pathology, the preservation of brain volume, and an improvement in behavioural scores [185]. Among the most relevant anti-tau vaccines is AADvac1, an active peptide vaccine targeting nonphosphorylated tau, which proved safe and immunogenic in AD patients [186], although there were no clinical effects in the whole cohort. Regarding passive immunization, three studies have been completed, but the results are still unpublished (BIIB092/gosuranemab, a humanised monoclonal antibody that binds to N-terminal tau (NCT03352557); RO7105705/semorinemab, an anti-tau IgG4 antibody (NCT02820896); and LY3303560/zagotenemab, a humanised anti-tau antibody derived from MCI-1) (NCT02754830). A trial on JNJ-63733657, a humanised monoclonal anti-tau antibody that binds to phosphorylated tau (NCT04619420), is in the recruiting phase. 

#### 3.1.3. Clinical Trials on α-syn Immunisation

Similar to that for Aβ, passive immunisation for synucleinopathies involves using different monoclonal antibodies against α-syn. In a mouse model of PD, antibodies against α-syn significantly attenuated the cognitive and motor deficits, with a consequent reduction in α-syn aggregates and pathological accumulations of soluble α-syn, total α-syn, and α-syn oligomers [187]. Building on the results in animal models, large clinical trials started. However, unfortunately, no positive results were obtained in one large trial on prasinezumab [188], observing no meaningful effect in global and imaging measures of PD progression and a large percentage of infusion reactions (up to 34% of patients). A better safety profile was obtained in a phase I trial on BIIB054 (cinpanemab) [189], but still without effects on clinical outcomes in the first year of the trial, leading to its discontinuation and premature termination (data not published). Clinical trials on α-syn active immunisation were conducted as a continuation of several studies of animal models, which showed that antibodies to α-syn prevented pathogenic protein spread and promoted clearance of aggregates [190]. Several human studies have been proposed. One of the most promising involved the use of PD01A (AFFITOPE). The molecule is an eight-amino-acid peptide that mimics an epitope in the C-terminal region of human α-syn designed to stimulate B-cell antibody responses bypassing the auto-reactive T-cell mobilisation. The first-in-human, randomised, phase 1 study on immunisations with PD01A [191] demonstrated that the repeated administrations of PD01A were safe and relatively well-tolerated in a cohort of PD patients. From a biological point of view, a substantial humoral immune response was observed. However, to date, no other results have been published.

#### 3.1.4. Immunisation for ALS Treatment

For ALS, monoclonal antibodies against intracellular proteins such as SOD1, C9orf72, and TDP-43 are under study or have been recently completed, although several improvements are needed to increase the efficacy in ALS patients and obtain significant results [192]. Other antibody-based interventions investigated in ALS patients target other molecules involved in the neuroinflammatory pathways mainly localised at the intracellular and extracellular levels, including the neurite outgrowth inhibitor A (ozanezumab), muscle-specific kinase, the IL-6 receptor (tocilizumab), and other proteins (namely NRP-1, myostatin, CD40L, DR-6, IFN-γ, GD1a, CTGF, and HMGB1). Also, in this case, the results are only preliminary, contrasting [193,194], and they are mainly related to biological outcomes [193].

### 3.2. Targeting Inflammatory Mediators

Although there are interesting data on the crosstalk of aggregated proteins in different models of NDDs, to date, immunotherapies targeting aggregates alone have had limited success. This leads us to assume that a more complex scenario exists in which the pathological proteins are only one facet of a much more complex therapeutic challenge. As already described, one of the most common and relevant targets of neurodegeneration is the alteration of CNS homeostasis mediated by the immune system, in which microglia and astrocytes play a central role. In the past years, several clinical trials using anti-inflammatory drugs (e.g., aspirin, prednisone, naproxen, diclofenac, and indomethacin) have been started in patients with NDDs, including AD, PD, and ALS, but all failed to obtain clinical improvements [195,196]. This suggests that the complexity between microglia, astrocytes, and neurodegeneration necessitates more precise targeting and perhaps action further upstream in the neuroinflammatory cascade, such as on inflammatory mediators (cytokines, chemokines, and growth factors) or phagocytic functions. However, converging targets with great cytolytic potential, such as the complement cascade, have also been targeted. For example, some potential targets are as follows. (1) TNF-α: the use of TNF-α inhibitors (antibodies and related fusion proteins) was promising in rodent models of AD; however, two previous studies in AD obtained contrasting results, mainly related to the difficulty of the drug (etanercept) to penetrate the CNS after perispinal injection [197]. Other routes of administration have been tried (e.g., subcutaneous), but with no relevant clinical results, probably related to its inability to penetrate the BBB [197]. (2) Interleukins: evidence reported that numerous interleukins (including IL-2, IL-17, and IL-22) are associated with NDD development and progression by activating glial cells and creating a pro-inflammatory environment. Preliminary evidence for using IL-2 in AD started from solid preclinical data [198], and some trials are currently ongoing (NCT05821153 and NCT05468073). (3) GM-CSF: GM-CSF is an immunomodulatory growth factor that is clearly deregulated in NDDs. GM-CSF has shown a strong positive effect in mouse models, showing a pleiotropic neuroprotective effect with the attenuation of neuroinflammation and cognitive decline by enhancing Aβ clearance by recruiting microglia to amyloid plaques [199]. The in-human results are also encouraging; a clinical trial published in 2021 on AD patients showed that GM-CSF (sargramostim) treatment had no adverse events in patients, changed innate immune system markers, and significantly improved cognitive status [200]. A larger trial is ongoing (NCT04902703). The same drug also showed encouraging effects in PD; even in a small pilot phase I clinical trial [201], sargramostim showed, clinically, a modest improvement after treatment compared with placebo, and biologically, a Treg number increase.

Regarding ALS, some anti-inflammatory therapies targeting the immune system have yielded promising results. Very recently, the group of Professor Mandrioli published interesting results on rapamycin treatment in ALS patients [202]. Specifically, the trial observed a significant decrease in the mRNA relative expression of IL-18, plasmatic IL-18 protein, and increased monocytes and memory-switched B cells. Other promising results were derived with an autologous infusion of expanded Tregs plus subcutaneous IL-2; Treg/IL-2 treatments promoted, from a biological point of view, a higher Treg suppressive function and, from a clinical point of view, a slowing in disease progression [203]. Lastly, a clinical trial with masitinib is underway (NCT03127267). Masitinib is a selective oral tyrosine kinase inhibitor with preclinically demonstrated neuroprotective effects and promising clinical effects in the ALS early trial stage. Interestingly, the same drug is also being studied in patients with mild to moderate AD (NCT05564169).

### 3.3. Targeting Complement

As shown, complement has pathogenic relevance in NDDs, in which the role of innate immune-driven inflammation is rapidly growing. Drug molecules that target players of the complement activation cascade can potentially stop complement-mediated tissue damage, and some trials are ongoing in this regard in NDDs [77]. For example, in AD mouse models, positive results in terms of safety have been obtained with ANX005 (a humanised immunoglobulin G4 recombinant antibody against C1q). No studies in humans are currently ongoing [204]. In ALS, similar results in mouse models were obtained using the PMX205, which could delay the grip decline and slow the disease progression [205]. Also, in ALS patients, two studies are ongoing targeting complement: a phase 2 trial on ANX005 (NCT04569435) and another phase 2 trial on pegcetacoplan (APL-2, a complement C3 inhibitor) (NCT04579666). Lastly, a phase 3 trial on ravulizumab (a long-acting inhibitor of terminal complement protein C5) was recently terminated but without a positive outcome (the internal displacement monitoring centre discontinued the study due to lack of efficacy). No trials on PD are ongoing.

### 3.4. Stem Cell and Related Treatments

Due to their immunomodulatory, anti-inflammatory, and regenerative properties, stem cells and particularly mesenchymal stromal cell (MSC) treatments can have long-term effects on patients with NDDs, as recently demonstrated in ALS [206,207]. From a scientific point of view, these positive results lay the foundations for continuing research in this field and applying it to other NDDs. MSCs (Lomecel-B) have recently been preliminary tested in AD in a phase I clinical trial [208] with positive results, including improvement in cognitive function, hippocampal tropism, and fluid biomarkers, providing mandatory information for larger phase II/III clinical trials. However, to date, there are no data about MSC transplantation in PD, but from one preliminary report, autologous bone marrow MSCs injected into the subventricular zone appeared safe and well-tolerated, with minimal motor function improvement [209,210].

Another important avenue in the treatment of NDDs is represented by exosomes. MSC-derived exosomes have been demonstrated to exert more potent therapeutic effects over MSCs in NDDs, principally by delivering anti-neuroinflammatory processes [209,210]. Currently, some clinical trials to test the efficacy of exosomes in AD (NCT0438 8982) and PD (NCY01860118) are ongoing.

### 3.5. Targeting Microbiota

With many of the pathways behind gut–brain signalling still waiting to be discovered, the main incentive for further research should be potential therapeutic methods targeting the gut microbiota. Antibiotics have shown a positive effect on PD pathology in mice, either by altering microbiota composition [211] or inhibiting α-syn fibrillation [212]. Similarly, probiotics have also been shown to prevent neuroinflammation and cognitive dysfunction by modifying microbiome composition [213]. Specific diets are known to have a beneficial effect in neurodegenerative diseases such as AD and PD, most notably the Mediterranean diet, which is rich in *Lactobacilli* [214]. Certain clinical studies have shown promise, such as in the case of enema application [215,216] or faecal microbiota transplantation [217]. In conclusion, since a great body of knowledge shows microbiome changes in NDDs, the gut, as an often-neglected organ in treating NDDs, should be taken into account. In time, microbiota regulation could prove to be a powerful tool for the prevention and management of neurodegenerative disorders.

### 3.6. Potential Future Therapies

Preventive vaccination and/or boosting for influenza virus, HSV-1, and herpes zoster virus have already been conclusively linked to a decreased risk of dementia [218,219]. Similar effects were reported for vaccines in preventing pneumococcal pneumonia. Although these vaccinations are still not a standard of care for adult populations in most countries, it is advisable that these notions are considered in future preventive measures for NDDs. Preventive and therapeutic measures will also likely involve microbiome alterations, but this work has still not advanced to the level to be systematically used. Senolytics, drugs that target senescent cells, have also been proposed as potential treatments for various NDDs, but their potential is not sufficiently explored [220,221].

Regarding PD, the primary limitation to advancing beyond dopamine replacement therapies has been that most patients are diagnosed well after significant neuropathology has occurred. As earlier biomarkers are developed, the likelihood of successful bench-to-bedside translation of novel interventions is expected to increase. As one promising example, a recent study found that mtDNA damage was increased in peripheral blood mononuclear cells derived from patients with idiopathic PD and those with the PD-associated leucine-rich repeat kinase 2 (LRRK2) G2019S mutation in comparison with age-matched controls. Importantly, mtDNA damage was elevated in non-disease-manifesting LRRK2 mutation carriers, suggesting that those with known risk factors but not yet with a PD phenotype (not all will convert) could be identified prior to diagnosis. Of note, LRRK2 plays a critical role in the central and systemic immune systems. These findings point to a broad future approach in which PD patients are identified earlier and may even be treated in the prodromal stages with specific immune modulators. Kinases linked to PD-relevant inflammatory responses have been an especially exciting target for early-stage interventions. Of significant note is LRRK2, which is highly expressed in monocytes and macrophages; PD-relevant mutant forms of this kinase increase cytokine production [222]. Overall, there is extensive evidence that LRRK2 kinase activity modulates PD-relevant neuroinflammatory responses [223,224,225]. Thus, the inhibition of LRRK2 kinase activity is an exciting experimental approach [225,226]. Given such promise, ongoing clinical trials are being conducted [227]. Overall, evolving literature showing that neuroinflammatory mechanisms are primary pathogenic pathways has led to therapeutic targeting of factors that drive PD-relevant neuroinflammation. Ultimately, the hope is that such therapies may be disease-modifying in terms of slowing or halting progression. Such treatments could be complementary to dopaminergic replacement therapies, which treat symptoms but do not alter progression.

Current treatments of NPC aim to lower the accumulation of free cholesterol and other GSLs in late endosomes/lysosomes—the primary feature of NPC disease. However, the use of miglustat or methyl-β-cyclodextrin, which inhibit cholesterol synthesis or reduce its accumulation, respectively, can alleviate symptoms somewhat but cannot sustainably halt the progression of the disease [228,229,230,231,232]. Given the overlap between AD and NPC pathology, including Aß plaque formation, tau accumulation, and early neuroinflammation (activation of microglia), future therapeutic options against NPC may benefit from those already tested in AD.

Finally, a large body of evidence from experimental animal models has pinpointed numerous highly specific inflammatory signalling targets that comprise potential new lines of treatment. Especially interesting are the cyclic GMP–AMP/stimulator of interferon genes (c-GAS/STING) pathway, inflammasomes, the NF-kB pathway, and the TANK-binding kinase 1/interferon regulatory 3 pathway (TBK1/IRF3) [233,234,235,236,237]. For example, the NLPR3 inflammasome could be an important target for NDDs and progressive MS [67,238]. Notably, all of these should be evaluated with caution, as translation from animal models of neurodegenerative disease is not always straightforward given the need for artificial overexpression of disease-causing human mutations or even simultaneous expression of several gain-of-function mutations [239,240], whereas numerous loss-of-function models exhibit mild or no phenotypes and do not phenocopy the course of human disease, as exemplified with TBK1 and optineurin disease models [237,241,242]. For these targets, it will likely be crucial to find an optimal window for neuroprotection because blockade of the signalling pathways could be as detrimental as their excessive activation. 

## 4. Conclusions

With their complex genetic and environmental aetiology and an ever-increasing incidence in modern society, NDDs remain one of the leading medical challenges. Given the extensive overlap between many adult-onset neurodegenerative diseases, particularly AD, PD, ALS, and FTD, it is encouraging that many of the potential immunosuppressive and immunomodulatory treatments directly targeting immune mediators have been studied across different NDDs. The prerequisites for moving forward were two paradigm shifts regarding immunity in NDDs: first, immune mechanisms are not merely noxious but also protective, and second, inflammation likely plays a role as a trigger that not only acts as a distal element but can also contribute to the early pathogenesis of the disease. The first resulted in a deeper understanding of the mechanisms and the design of more nuanced targeted therapies instead of using broadly acting immunosuppressants, whereas the second will in time perhaps allow us to focus more on prevention and vaccinations to mitigate the risk for NDDs. Common targets for immunomodulatory treatment are of specific interest, and it is encouraging that some have also shown promising results across the NDD spectrum (Tregs, complement, etc). An overlap of therapeutic approaches is also expected between NDDs and progressive MS. However, in the diversity of the NDD spectrum, even within the same diagnostic entity, it is hard to imagine that one target treatment could be curative. More likely, it will be necessary to apply drug cocktails targeting different mechanistic pathways involved in the disease processes, necessitating a deeper understanding of the disease mechanisms. From a bird’s-eye view, drawing a line through the common denominators of NDDs, we might find a way to explore new mechanistic treatment targets that will be beneficial to a spectrum of clinically completely different NDDs. In conclusion, given the multifactorial nature, the numerous disease mechanisms, and the overlapping proteinopathies of the different NDDs, combining treatments acting on different disease pathways may allow an integrated and synergic disease management intervention, personalised to individual patients.

## Data Availability

Not applicable.
